# Supplementary material for: Integrative Multi-Omics Elucidates the Therapeutic Effect of Coix Seed Oil on Rheumatoid Arthritis via the Gut-Butyrate-Joint Axis and NLRP3 Inflammasome Suppression
Source: Pharmaceuticals (Basel). 2025 Dec 25;19(1):48. doi: 10.3390/ph19010048 (PMC12844696; doi:10.3390/ph19010048)
Supplement: Supplementary file 1 [file pharmaceuticals-19-00048-s001.zip › pharmaceuticals-3996907-supplementary.pdf]

# Supplementary Materials

A

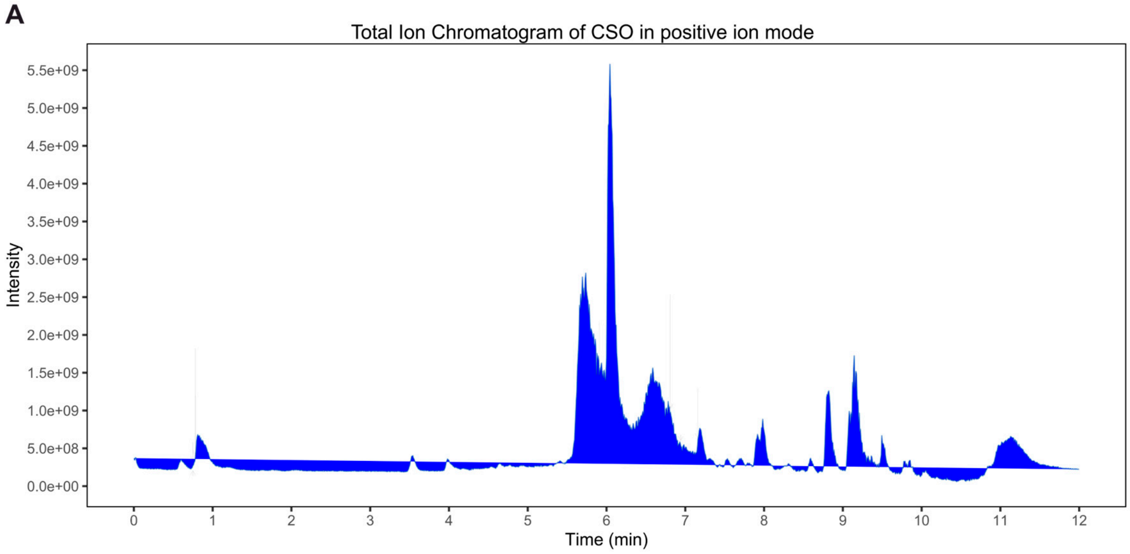


B

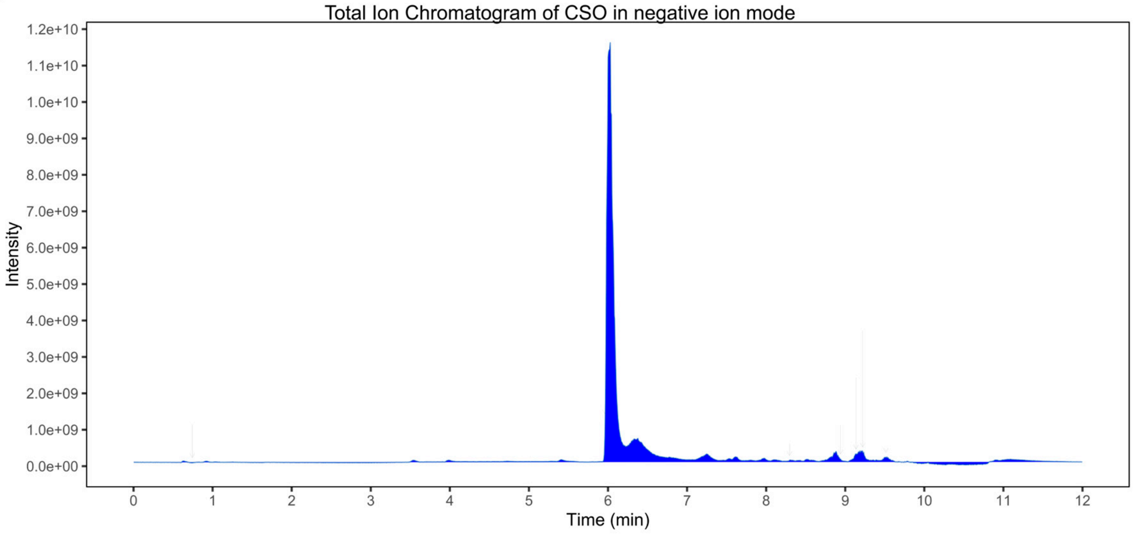


**Figure S1.** (**A**) Base peak ion current chromatogram of the CSO detected in positive ion mode. (**B**) Base peak ion current chromatogram of the CSO detected in negative ion mode.
